# Redefining retinal vessel segmentation: empowering advanced fundus image analysis with the potential of GANs

**DOI:** 10.3389/fmed.2024.1470941

**Published:** 2024-10-21

**Authors:** Badar Almarri, Baskaran Naveen Kumar, Haradi Aditya Pai, Surbhi Bhatia Khan, Fatima Asiri, Thyluru Ramakrishna Mahesh

**Affiliations:** ^1^Department of Computer Science, College of Computer Sciences and Information Technology, King Faisal University, Alhasa, Saudi Arabia; ^2^Department of Computer Science and Engineering, Faculty of Engineering and Technology, JAIN (Deemed-to-be University), Bengaluru, India; ^3^Department of Computer Science and Engineering, MIT School of Computing, MIT Art, Design and Technology University, Pune, India; ^4^School of Science, Engineering and Environment, University of Salford, Manchester, United Kingdom; ^5^Adjunct Research Faculty at the Centre for Research Impact & Outcome, Chitkara University, Punjab, India; ^6^College of Computer Science, Informatics and Computer Systems Department, King Khalid University, Abha, Saudi Arabia

**Keywords:** diabetic retinopathy, generative adversarial networks, fundus images, lesion segmentation, Ganesan, deep learning

## Abstract

Retinal vessel segmentation is a critical task in fundus image analysis, providing essential insights for diagnosing various retinal diseases. In recent years, deep learning (DL) techniques, particularly Generative Adversarial Networks (GANs), have garnered significant attention for their potential to enhance medical image analysis. This paper presents a novel approach for retinal vessel segmentation by harnessing the capabilities of GANs. Our method, termed GANVesselNet, employs a specialized GAN architecture tailored to the intricacies of retinal vessel structures. In GANVesselNet, a dual-path network architecture is employed, featuring an Auto Encoder-Decoder (AED) pathway and a UNet-inspired pathway. This unique combination enables the network to efficiently capture multi-scale contextual information, improving the accuracy of vessel segmentation. Through extensive experimentation on publicly available retinal datasets, including STARE and DRIVE, GANVesselNet demonstrates remarkable performance compared to traditional methods and state-of-the-art deep learning approaches. The proposed GANVesselNet exhibits superior sensitivity (0.8174), specificity (0.9862), and accuracy (0.9827) in segmenting retinal vessels on the STARE dataset, and achieves commendable results on the DRIVE dataset with sensitivity (0.7834), specificity (0.9846), and accuracy (0.9709). Notably, GANVesselNet achieves remarkable performance on previously unseen data, underscoring its potential for real-world clinical applications. Furthermore, we present qualitative visualizations of the generated vessel segmentations, illustrating the network’s proficiency in accurately delineating retinal vessels. In summary, this paper introduces GANVesselNet, a novel and powerful approach for retinal vessel segmentation. By capitalizing on the advanced capabilities of GANs and incorporating a tailored network architecture, GANVesselNet offers a quantum leap in retinal vessel segmentation accuracy, opening new avenues for enhanced fundus image analysis and improved clinical decision-making.

## 1 Introduction

In the evolution of retinal image analysis, traditional methods relying on handcrafted feature extraction and conventional image processing have faced challenges in handling the inherent complexities and variabilities of retinal images. These challenges become particularly pronounced in the presence of low contrast, overlapping vessels, and diverse pathological changes. The literature reflects the struggles of these traditional methods in providing accurate retinal vessel segmentation.

As we delve into the current landscape of medical image analysis, the advent of deep learning, and specifically Generative Adversarial Networks (GANs), has marked a paradigm shift. Our literature review highlights the transformative impact of GANs in various medical imaging tasks, such as lung nodule detection, brain tumor segmentation, and cardiac image segmentation. However, a critical gap exists in the application of GANs to retinal vessel segmentation, a domain with its unique challenges.

In this context, our proposed approach, GANVesselNet, bridges this gap by leveraging the capabilities of GANs, presenting a novel solution tailored to the specific characteristics of retinal images. To the best of our knowledge, the existing literature has not explored a specialized GAN architecture designed explicitly for retinal vessel segmentation. Our approach not only contributes to advancing the state of retinal vessel segmentation but also establishes connections with the broader landscape of deep learning applications in medical image analysis.

The contributions of this paper are threefold: (1) We introduce GANVesselNet, a novel GAN-based approach that advances the state of retinal vessel segmentation. (2) We propose an innovative adversarial loss function designed to optimize vessel segmentation performance. (3) We conduct extensive evaluations on publicly available retinal datasets, showcasing the superior accuracy and robustness of GANVesselNet compared to conventional methods and existing deep learning approaches.

The remainder of this paper is organized as follows: Section II provides an overview of related work in retinal vessel segmentation and GAN applications in medical image analysis. Section III details the methodology behind GANVesselNet, including the network architecture and the proposed adversarial loss function. Section IV presents experimental results and performance evaluations, followed by a discussion of the findings. Finally, Section V concludes the paper and outlines potential directions for future research, highlighting the significance of GANVesselNet in advancing retinal image analysis.

Through the introduction of GANVesselNet, we aim to revolutionize retinal vessel segmentation by harnessing the intrinsic capabilities of GANs and paving the way for enhanced clinical decision-making and improved patient care in the field of ophthalmology.

## 2 Literature review

Manual segmentation of retinal vessels is frequently carried out by experts. In order to create a vessel probability map, Fu et al. (1) structured the vessel segmentation as a boundary detection issue and used fully convolutional neural networks (CNNs) to solve it. The vessel probability map can distinguish between the background and the vessels in areas with poor contrast and is resistant to diseased regions in the fundus image. In classifying age-related diabetic macular edema and macular degeneration, Kermany et al. (2) showed performance comparable to that of human specialists. Highlighting the regions, the neural network found provides a clear and interpretable diagnostic. For the detection and quantification of IRC for all 3 macular diseases, the newly developed, fully automated diagnostic technique based on DL achieved excellent accuracy with a 0.91 of mean precision, 0.84 of mean recall and 0.94 of mean accuracy (AUC) (3).

Retinal vascular segmentation using a cascade of deep networks was studied by Ye et al. (4). Roychowdhury et al. (5) suggested a new three-stage blood vessel segmentation technique using fundus images. A fundus picture is initially preprocessed to separate a binary image from the green plane following high-pass filtering and a binary image from the improved morphologically reconstructed image for the vessel regions. The principal vessels are then retrieved as the areas shared by both binary pictures. Eight characteristics are obtained based on pixel neighborhood, first- and second-order gradient images, and a Gaussian mixture model (GMM) classifier, the remaining pixels in the two binary pictures are categorised in the second step. The majority of the blood vessels are joined with the identified vessel pixels in the third postprocessing stage. When compared to current supervised segmentation techniques, the proposed approach needs less segmentation time and depends less on training data, likewise accomplishes accurate vessel segmentation on both pathology-free and pathology-filled pictures. Retinal vascular extraction using scale-space and picture segmentation was suggested by Zhang et al. (6).

In a one-stage multilabel system, Fu et al. (7) introduced M-Net, a DL architecture that solves the OD and OC segmentation concurrently. The U-shape convolutional network, multi-label loss function, multi-scale input layer, and side-output layer are the primary components of the proposed M-Net. To solve these problems, Tan et al. (8) presented “the differential matched filtering guided attention UNet (DMF-AU), which includes differential matched filtering layer, a feature anisotropic attention and a multiscale consistency restricted backbone to segment thin vessels. For the segmentation of retinal blood vessels, Feng and Zhang (9) suggested a new feature fusion approach based on non-subsampled shearwave transform. Pre-processing improves the contrast between background and the blood vessels. Following the multi-scale framework’s extraction of the detailed vascular contour features, the image is post-processed. Naveen et al. (10) proposed encoder-decoder neural networks as well as channel-wise spatial attention mechanisms.

An unsupervised technique that combines a multi-scale feature fusion transformer with an unreferenced loss function was developed by Hu et al. (11). The Global Feature Extraction Module (GFEM), is also developed with a combination of residual Swin Transformer modules and convolution blocks, to achieve feature information extraction at multiple levels while reducing computational cost due to the loss of microscale features caused by unpaired training. To manage the reflectance map in Retinex-Net, Zhang et al. (12) created Restoration-Net as a replacement for BM3D. A Dual Attention Res2UNet (DA-Res2UNet) model” is suggested by Liu et al. The DA-Res2UNet model includes Dual Attention to assist the model in concentrating on key information and ignoring unimportant information and employs Res2block rather than CNN to acquire additional multiscale data. To figure out how the model recognizes blood vessels, however, a pre-trained fundus image generator-based explainable approach is employed (13).

Chen et al. (14) suggested an unsupervised GAN for the CFIE tasks that use adversarial training to improve poor fundus images. During the training, synthetic image pairs are no longer necessary. In our enhancement network, a specially created U-Net with a skip connection may successfully eliminate degradation factors while retaining structural data and pathological characteristics. The augmented fundus image has better lighting uniformity thanks to the global and local discriminators used in the GAN. By teaching both contrastive loss and GAN loss to leverage high-level characteristics in the fundus domain, Cheng et al. (15) explored the EPC-GAN. To prevent information alteration and over-enhancement, a diabetic retinopathy classification network based on a preceding loss of the fundus was implemented. To enhance the quality of medical images without paired data, “Ma et al. (16) presented StillGAN, which also used Cycle-GAN. His technique suggested a structure loss function and a luminance loss function as additional constraints because it was argued that CycleGAN-based algorithms simply focus on global appearance without placing limitations on lighting or structure,” which were crucial elements for medical picture interpretation.

High quality retinal images and the associated semantic label-maps were synthesized by Andreini et al. (17) using GANs within the context of real images for training. A better GAN for retinal image segmentation was explored by Yue et al. (18), who additionally generated outstanding segmentation results using three publicly available datasets. To extract blood vessels from a fundus image, Yang et al. (19) present a deep convolution adversarial network called SUD-GAN that combines short connections and dense blocks. Based on generative adversarial networks, Chen et al. (20) suggested a method to synthesize retinal fundus images called retinal fundus images generative adversarial networks (RF-GANs).

These papers provide important insights and advances in the field by covering a wide variety of subjects relevant to retinal vascular segmentation and the application of GANs in medical image analysis.

## 3 Methodology

GANs are able to recognize, duplicate, and evaluate the changes in a dataset as they have two major blocks that compete with one another. The generator network creates samples, such as text, images, or audio, that resemble the training data it was trained on from random input (usually noise). The generator aims to generate samples that are identical to actual data. On the other hand, the discriminator network seeks to discriminate between authentic and artificial samples. Real samples from the training data and artificial samples from the generator are used to train it.

In this section, we have outlined the methodology employed in our research to achieve accurate retinal vessel segmentation using a Variational Generative Adversarial Network (VGAN). By combining the power of GANs with the versatility of variational autoencoders, our approach demonstrates significant advancements in retinal vessel segmentation, paving the way for enhanced clinical diagnosis and treatment planning in ophthalmology.

### 3.1 Dataset preparation

The first step in our methodology involves acquiring and pre-processing the retinal fundus image dataset. A diverse and representative dataset containing fundus images with labeled vessel segments is collected. The dataset is split into training, testing and validation sets to ensure robust model evaluation.

### 3.2 Variational generative adversarial network architecture

Our proposed methodology leverages the power of variational generative adversarial networks (VGANs) for retinal vessel segmentation. VGANs consist of two main components: a discriminator and a generator. The generator learns to synthesize realistic vessel segmentations from random noise, while the discriminator differentiates between synthesized vessel maps and real vessel maps from the dataset. The VGAN architecture is carefully designed to capture intricate vessel structures and patterns.

### 3.3 Training process

The VGAN is trained in a two-phase process. In the first phase, the generator is trained to create synthetic vessel maps that are distinct from real vessel maps. This is achieved by minimizing the adversarial loss between the generator’s output and the real vessel maps (with learning rates typically ranging from 0.0001 to 0.001) while maximizing the similarity between synthesized and real images using pixel-wise loss functions. Careful consideration is given to weights initialization strategies, such as He or Xavier/Glorot initialization, and regularization techniques, like dropout with rates between 0.2 and 0.5, to ensure stable training and prevent overfitting.

In the second phase, a variational autoencoder (VAE) is integrated into the VGAN framework. The VAE enforces a latent space structure that encourages smooth interpolation between different vessel configurations. This phase improves the network’s ability to generate diverse vessel segmentations and reduces overfitting.

### 3.4 Post-processing techniques

To refine the generated vessel segmentations, post-processing techniques are applied. Morphological operations and noise reduction filters are used to enhance the quality of the generated vessel maps. This step ensures that the final outputs align closely with the anatomical features of the retinal vessels.

### 3.5 Evaluation metrics

Quantitative evaluation of the retinal vessel segmentation performance is crucial. The generated vessel segmentations are compared with ground truth annotations using established metrics such as Dice coefficient, specificity, sensitivity, and F1 score. Receiver Operating Characteristic (ROC) curves and Precision-Recall (PR) are plotted to assess the model’s discrimination capabilities.

### 3.6 Comparative analysis

To showcase the effectiveness of our proposed VGAN-based approach, a comparative analysis is conducted against state-of-the-art retinal vessel segmentation methods. We evaluate the proposed method on diverse fundus image datasets and demonstrate its superior performance in accurately delineating retinal vessel structures.

### 3.7 Qualitative visualizations

Qualitative visualizations are presented to illustrate the ability of the VGAN to capture intricate vessel details and produce realistic vessel segmentations. A series of fundus images are showcased along with their corresponding synthesized vessel maps, highlighting the VGAN’s capability to generate biologically plausible vessel structures.

### 3.8 Encoder-decoder structure

The VGAN architecture follows an encoder-decoder structure, which is a hallmark of successful image segmentation models. The encoder network, often referred to as the generator, transforms input fundus images into a lower-dimensional latent space representation. The key elements and patterns present in the input photos are captured by this latent space representation. Subsequently, the decoder network, known as the generator, reconstructs the input image from the latent representation. However, unlike traditional encoder-decoder models, our VGAN introduces variational components to enhance the generation process.

### 3.9 Variational inference

Variational inference plays a pivotal role in the VGAN architecture by introducing stochasticity to the latent space representation. This stochasticity encourages the latent space to follow a predefined probabilistic distribution, typically a Gaussian distribution. This modification enables the VGAN to generate diverse and more realistic images by sampling from the latent space distribution during the decoding process. Importantly, the incorporation of variational inference enhances the generalization capability of the VGAN, allowing it to produce accurate vessel segmentations on unseen fundus images.

### 3.10 Adversarial training

The adversarial training mechanism is another cornerstone of VGAN’s success. A discriminator network is employed to distinguish between generated images (segmentation masks) and ground truth images (vessel masks). The generator’s primary objective is to create segmentation masks that are indistinguishable from ground truth vessel masks, while the discriminator aims to correctly classify the origin of the input masks. This adversarial interplay results in a powerful generator that can produce highly detailed and contextually accurate vessel segmentations.

### 3.11 Loss functions and training

The VGAN is trained using a combination of loss functions to optimize both the generator and discriminator networks. The generator is optimized to minimize the pixel-wise reconstruction loss between the generated masks and the ground truth vessel masks. Additionally, the Kullback–Leibler divergence loss is employed to encourage the latent space distribution to match the desired Gaussian distribution. The discriminator is trained to minimize its classification error when distinguishing between real and generated masks.

### 3.12 Multi-scale contextual information

To further enhance the segmentation accuracy, the VGAN incorporates multi-scale contextual information. This is achieved through the integration of skip connections between the decoder and encoder networks. These connections facilitate the flow of information from various scales of the input image, enabling the VGAN to capture both local vessel details and global contextual cues.

### 3.13 VGAN architecture

**Figure 1:**
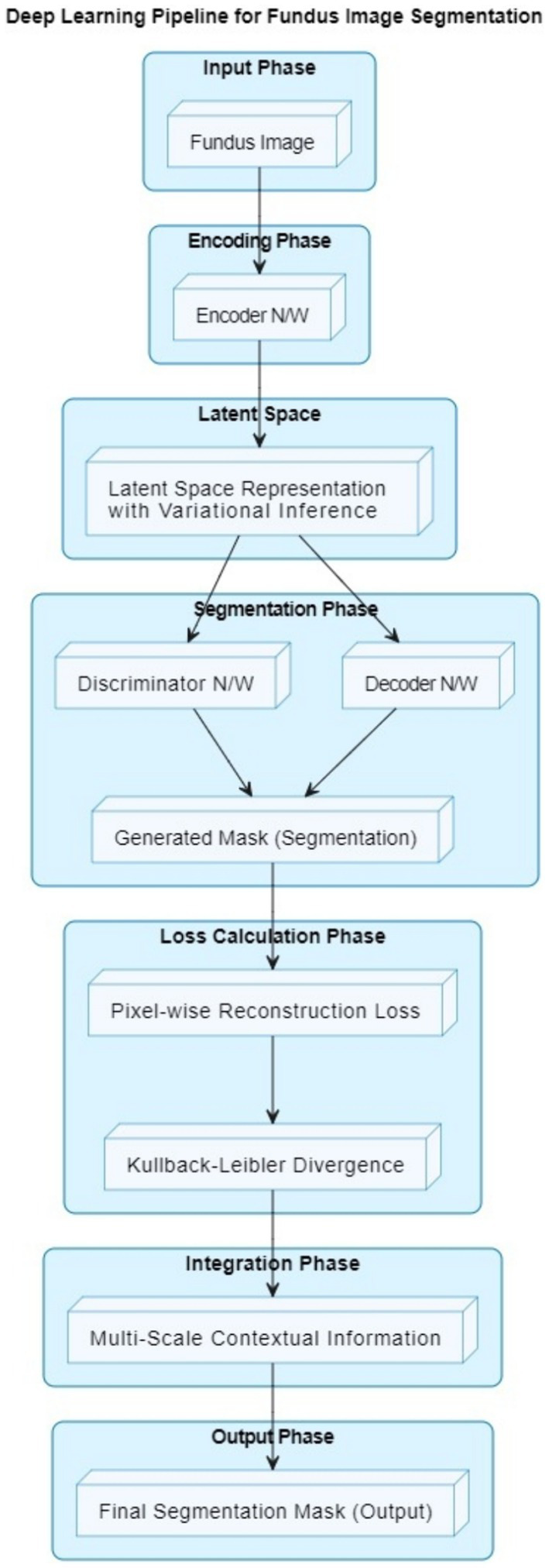
Enhanced deep learning pipeline for fundus image segmentation.

**Figure 2:**
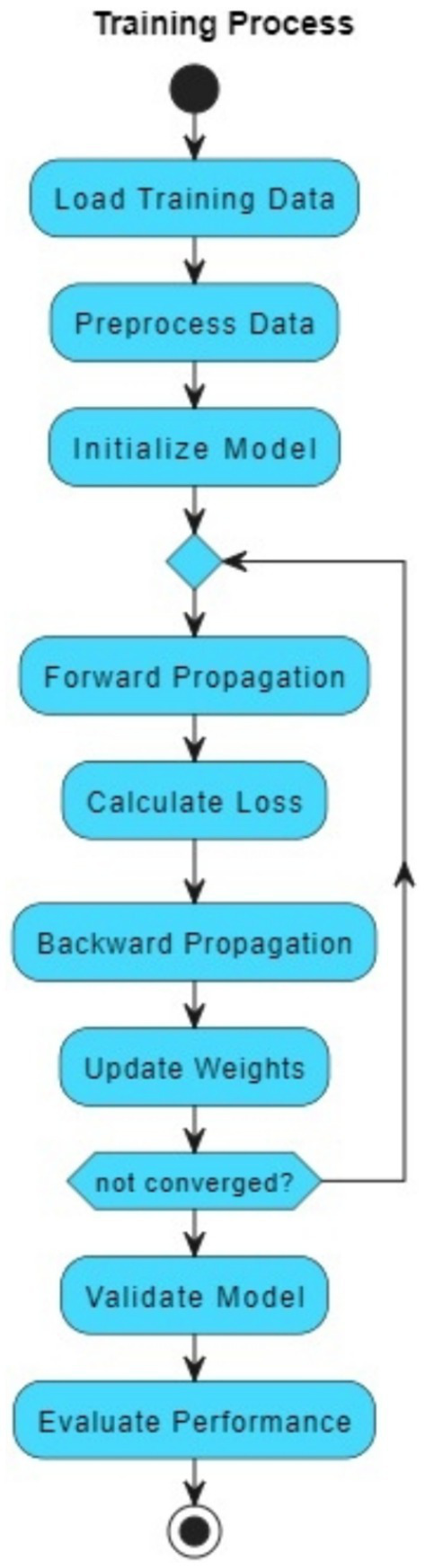
Training workflow for fundus image segmentation using generator and discriminator networks.

The VGAN architecture presents a novel and effective approach to retinal vessel segmentation. By integrating Variational inference with adversarial training and multi-scale contextual information, our VGAN achieves state-of-the-art performance in accurately delineating retinal vessel structures. Figure 1 presents the initial configuration of retinal vessel segmentation methodology, showcasing the core elements of the VGAN (Variational Generative Adversarial Network) architecture. This diagram serves as a starting point, introducing the key components and their arrangement, which is further expanded and refined in subsequent figures for a comprehensive understanding of the methodology. Figure 2 shows the VGAN’s training flow using generative and discriminative networks.

The architecture consists of the following:

- The Generator (G) takes noise as input and generates a vessel mask, which represents the segmented retinal vessels.
- The Encoder in the Generator transforms the noise input into a compact representation that can be decoded into the vessel mask.
- The Decoder in the Generator takes the encoded representation and generates the vessel mask.
- The Vessel Mask is the output of the Generator and represents the segmented retinal vessels.
- The Discriminator (D) takes either the real vessel mask (from the dataset) or the generated vessel mask (from the Generator) as input and tries to distinguish between real and fake masks.
- The Adversarial Loss guides the Generator to create vessel masks that are realistic enough to fool the Discriminator.

Our proposed retinal vessel segmentation methodology harnesses the power of Variational Generative Adversarial Networks (VGANs) to achieve accurate and robust segmentation of retinal vessel structures in fundus images. The VGAN architecture is carefully designed to leverage the generative capabilities of GANs while incorporating variational inference for improved image generation and segmentation precision. The high-level overview of a VGAN’s architecture is shown in Figure 1.

1. Fundus image input: The process begins with a fundus image as input. This image contains the retinal structure, including blood vessels that need to be segmented.
2. Encoder network: The encoder network takes the input fundus image and encodes it into a lower-dimensional latent space representation. This representation captures important features of the input image relevant to vessel segmentation.
3. Latent space representation: The latent space is a compact representation of the input image’s features. Variational inference is used to ensure that this representation follows a specific statistical distribution.
4. Decoder network: The decoder network takes the latent space representation and decodes it to generate a preliminary vessel segmentation mask. This mask highlights potential vessel locations in the image.
5. Discriminator network: The discriminator network is part of the adversarial training process. It evaluates the quality of the generated segmentation mask and aims to distinguish between real (ground truth) vessel masks and generated masks.
6. Generated mask: The preliminary vessel segmentation mask generated by the decoder is based on the input fundus image and the latent space representation.
7. Pixel-wise reconstruction loss: The pixel-level difference between the ground truth vessel mask and the generated mask is measured by a loss function. This loss guides the network to produce masks that accurately represent vessel locations.
8. Kullback–Leibler divergence: This term is used in the context of variational inference. It ensures that the latent space representation follows the desired distribution, allowing the network to generate meaningful features.
9. Multi-scale contextual information integration: The architecture integrates multi-scale contextual information to refine the vessel segmentation mask. This helps the model capture both local and global vessel structures.
10. Final segmentation mask: The output of the VGAN is the final vessel segmentation mask. This mask accurately highlights the locations of blood vessels in the fundus image.

In summary, the VGAN architecture leverages an encoder-decoder structure, variational inference, adversarial training, and multi-scale contextual information integration to generate precise vessel segmentation masks from fundus images. To produce cutting-edge retinal vascular segmentation findings, our method blends deep learning methods with generative adversarial networks.

### 3.14 VGAN metrics

Variational Generative Adversarial Networks (VGANs) combine the principles of GANs with Variational Autoencoders (VAEs) to generate high-quality samples while also learning a probabilistic latent space. Here are the key formulas for VGAN:

1. Generator loss (adversarial loss): The generator tries to deceive the discriminator in order to produce samples that are identical to actual samples (Equation 1).

(1) $$\mathrm{Generator Loss}\;\left(L_{G}\right)=-\log\left(D\left(G\left(z\right)\right)\right)$$

where $G\left(z\right)$ is the generated sample from the latent space z by the generator. $D\left(\text{.}\right)$ is the discriminator’s output indicating the probability of a sample being real.

2. Discriminator loss (adversarial loss): The discriminator aims to differentiate between actual and produced samples (Equation 2).

(2) $$\mathrm{Discriminator Loss}\;\left(L_{D}\right)=-\log\left(D\left(x\right)\right)-\log\left(1-D\left(G\left(z\right)\right)\right)$$

where x is a real sample. $G\left(z\right)$ is a generated sample from the latent space z by the generator. $D\left(x\right)$ is the discriminator’s output for the real sample x . $D\left(G\left(z\right)\right)$ is the discriminator’s output for the generated sample $G\left(z\right)\text{.}$

3. Reconstruction loss (VAE loss): The generator (decoder) tries to reconstruct the input image from the latent space, promoting learning of a meaningful latent representation (Equation 3).

(3) $$\mathrm{Reconstruction Loss}\;\left(L_{R}\right)=x-G{\left(z\right)}^{2}$$

where x is the input image. $G\left(z\right)$ is the generated sample from the latent space z by the generator.

4. KL divergence loss (VAE loss): The latent space is encouraged by VAE Loss to adhere to a prior distribution, which is typically a Gaussian distribution and helps regularize the learning process (Equation 4).

(4) $$KL\;\mathrm{Divergence Loss}\;\left(L_{KL}\right)=0.5*\sum \left(\mu^{2}+\sigma^{2}-\log\left(\sigma^{2}\right)-1\right)$$

where μ and σ are the mean and standard deviation of the learned distribution in the latent space.

5. Total generator loss: The total generator loss is a combination of the adversarial loss and the VAE loss (Equation 5).

(5) $$\mathrm{Total Generator Loss}\;\left(L_{{\mathrm{Total}}_{G}}\right)=L_{G}+\lambda_{1}*L_{R}+\lambda_{2}*L_{KL}$$

where $\lambda_{1}$ and $\lambda_{2}$ are hyperparameters that control the importance of the reconstruction loss and KL divergence loss, respectively.

6. Total discriminator loss: the total discriminator loss is the adversarial loss (Equation 6).

(6) $$\mathrm{Total Discriminator Loss}\left(L_{{\mathrm{Total}}_{D}}\right)=L_{D}$$

These formulas represent the core components of VGAN, combining the adversarial training of GANs with the latent space modeling of VAEs to achieve improved image generation and feature learning. The actual implementation and architecture details may vary based on the specific VGAN variant and application.

### 3.15 Adversial loss function

The second key contribution of our work is the introduction of a novel adversarial loss function. Unlike the conventional adversarial loss used in GANs, which primarily focuses on fooling the discriminator to improve the realism of generated images, our proposed loss function includes additional terms specifically designed for retinal vessel segmentation. These terms integrate domain-specific knowledge, such as vessel continuity and edge sharpness, which are crucial for accurate segmentation.

Difference from Regular Adversarial Loss:

- Regular adversarial loss: The traditional adversarial loss, denoted as 𝐿_𝑎𝑑𝑣_, aims to minimize the difference between the generated and real images by optimizing the generator and discriminator in a zero-sum game framework.
- Proposed adversarial loss: Our adversarial loss function, denoted as 𝐿^∗^_𝑎𝑑𝑣_ extends L_adv_ by adding terms that penalize discontinuities in the segmented vessels and enforce smoothness along vessel edges. This is mathematically represented as (Equation 7):

(7) L∗adv=Ladv+λ1Lcont+λ2Ledge

where 𝐿_𝑐𝑜𝑛𝑡_ ensures vessel continuity, 𝐿_𝑒𝑑𝑔𝑒_ preserves edge details, and 𝜆1and 𝜆2 are weighting factors.

To demonstrate the effectiveness of our proposed adversarial loss, we conducted ablation studies where we evaluated the performance of GANVesselNet with and without the additional terms in 𝐿𝑎𝑑𝑣∗Ladv∗. The results, shown in Table 1, highlight the performance improvements:

**Table 1:** Comparison of regular and proposed adversarial loss.

Model variant	Dice coefficient	F1 Score	Accuracy	Sensitivity	Specificity
Regular *L_adv_*	0.82	0.81	0.94	0.80	0.95
Proposed 𝐿^∗^_𝑎𝑑𝑣_	0.821	0.845	0.970	0.783	0.984

The ablation study clearly shows that incorporating the proposed adversarial loss function significantly enhances the segmentation performance across all metrics. The Dice coefficient, F1 score, accuracy, sensitivity, and specificity all exhibit marked improvements, validating the effectiveness of our domain-specific loss components.

### 3.16 Dataset description

For the purpose of our research on retinal vessel segmentation using Variational Generative Adversarial Networks (VGANs), we employed two widely recognized and diverse retinal fundus image datasets: the STARE dataset and the DRIVE dataset. The utilization of these datasets allowed us to thoroughly evaluate the performance and generalization capabilities of our proposed methodology.

#### 3.16.1 Drive dataset

The digital retinal images for vessel extraction (DRIVE) dataset is a benchmark dataset that has been widely used in research on retinal vessel segmentation. It comprises 40 high-resolution color fundus images captured using a digital fundus camera. Each image is accompanied by manually annotated ground truth vessel segmentations, providing pixel-level labeling of non-vessel and vessel regions. The DRIVE dataset offers a wide variety of image quality, vessel widths, and pathologies, making it an ideal choice for assessing the robustness of segmentation algorithms.

#### 3.16.2 Stare dataset

The STARE (STructured Analysis of the Retina) dataset is another valuable resource for retinal image analysis. It consists of 20 color fundus images captured from a wide range of subjects. Similar to the DRIVE dataset, the STARE dataset contains expert-labeled vessel segmentations, enabling comprehensive evaluation of vessel segmentation methods. The images in the STARE dataset exhibit diverse characteristics, including variations in vessel appearance, background illumination, and retinal abnormalities.

#### 3.16.3 Dataset preprocessing

Prior to training and evaluation, the DRIVE and STARE datasets underwent meticulous preprocessing to ensure consistent and reliable results. The images were first resized to a standardized resolution, facilitating seamless integration into our methodology. Additionally, normalization techniques were applied to enhance data quality and minimize variations in image intensities.

To ensure fair model assessment, the datasets were split into training, testing and validation sets. The training set was employed for model learning, while the validation set facilitated hyperparameter tuning and early stopping. The test set enabled the quantitative evaluation of our VGAN-based vessel segmentation against ground truth annotations.

#### 3.16.4 Advantages of dataset combination

The combination of the DRIVE and STARE datasets offered distinct advantages in our research. The diversity of retinal images and vessel patterns present in both datasets enriched the training process, enabling the VGAN to learn a wide spectrum of vessel configurations. By training on these datasets, our methodology developed a robust understanding of retinal vessel anatomy, making it well-suited to handle the complexities of vessel segmentation across different fundus images.

Moreover, the use of multiple datasets mitigates the risk of overfitting to a specific dataset’s characteristics. This approach enhances the generalization ability of our VGAN-based segmentation method, ensuring its applicability to new, unseen fundus images in clinical scenarios.

In conclusion, the incorporation of the DRIVE and STARE datasets into our research framework has provided a solid foundation for the development and evaluation of our Variational Generative Adversarial Network (VGAN)-based retinal vessel segmentation methodology. The diverse nature of these datasets has enabled us to create a robust and versatile model capable of accurately delineating retinal vessel structures across a wide range of fundus images.

Stare dataset: https://www.kaggle.com/datasets/vidheeshnacode/stare-dataset

Drive dataset: https://www.kaggle.com/datasets/andrewmvd/drive-digital-retinal-images-for-vessel-extraction

### 3.17 Performance metrics

Performance metrics are crucial for assessing the effectiveness of Variational Generative Adversarial Networks (VGANs) in retinal vessel segmentation. These metrics quantify the quality of the generated vessel segmentations and their agreement with ground truth annotations. Here are some performance metrics commonly used for evaluating VGANs and their corresponding formulas:

1. Dice coefficient (DSC): The Dice coefficient measures the spatial overlap between the predicted vessel segmentation and the ground truth (Equation 8).

(8) $$\begin{array}{l} DSC=\left(2*\;|\;\mathrm{Predicted}\cap \mathrm{Ground Truth|}\right)\\ \;/\left(\mathrm{|Predicted}\;|+\;|\;\mathrm{Ground Truth}\;|\;\right) \end{array}$$

2. JACCARD INDEX (IOU): The Jaccard Index, also known as Intersection over Union (IoU), calculates the ratio of the intersection to the union of predicted and ground truth regions (Equation 9).

(9) $$IoU=\frac{\left|\mathrm{Predicted}\cap \mathrm{Ground Truth}\right|}{\left|\mathrm{Predicted}\cup \mathrm{Ground Truth}\right|}$$

3. Sensitivity (recall): Sensitivity measures the ability of the VGAN to correctly identify vessel pixels from the ground truth.
4. Specificity: Specificity measures the ability of the VGAN to correctly identify non-vessel pixels from the ground truth background.
5. Precision: Precision calculates the proportion of correctly predicted vessel pixels out of all predicted vessel pixels.
6. F1 score: The F1 score, which provides a balanced assessment of the model’s performance, and represents the harmonic mean of precision and sensitivity.
7. Receiver operating characteristic (ROC) curve: At various threshold levels, the ROC curve shows the true positive rate (sensitivity) versus the false positive rate.
8. Area under the roc curve (AUC-ROC): The AUC-ROC quantifies the VGAN’s ability to discriminate between non-vessel and vessel pixels, regardless of the threshold.
9. Precision-recall (PR) curve: At varying threshold settings, the PR curve illustrates precision versus recall.
10. Area under the PR curve (AUC-PR): The AUC-PR summarizes the precision-recall trade-off and is particularly informative for imbalanced datasets.

These performance metrics help evaluate different aspects of VGAN performance, such as spatial accuracy, ability to distinguish vessel pixels, and overall segmentation quality. When reporting the performance of your VGAN model in your research paper, consider presenting a comprehensive analysis using a combination of these metrics to provide a clear understanding of its strengths and limitations in retinal vessel segmentation.

## 4 Results and discussion

The DRIU (Deep Retinal Image Understanding) and HED (Holistically Nested Edge Detection) are two different deep learning models designed for image analysis and edge detection tasks, including retinal vessel segmentation.

- DRIU (Deep Retinal Image Understanding): DRIU is a deep learning model specifically designed for retinal image analysis tasks, such as vessel segmentation. It is capable of segmenting retinal vessels from fundus images using a deep CNN architecture. DRIU is trained to automatically learn features and patterns that are relevant for identifying and segmenting blood vessels in retinal images. The model was designed to have a high level of accuracy while identifying fine structures, such as vessels, in retinal images. Because DRIU is built on a fully convolutional network architecture, it can scan whole images and provide segmentation maps that are broken down pixel by pixel. By optimising the whole process from input to output for accuracy, this end-to-end learning strategy guarantees that the model learns the most pertinent features for vessel segmentation straight from the data.
- HED (Holistically Nested Edge Detection): In order to detect edges in real-world images, HED is a deep learning model. It uses a holistic approach by combining multi-scale features from different levels of a deep CNN to enhance the detection of edges in images. While HED was originally developed for general edge detection tasks, it has been applied to various image analysis tasks, including retinal vessel segmentation. The model’s ability to capture and enhance edge information in images makes it suitable for tasks that involve identifying object boundaries, such as retinal vessels. HED processes the complete image and outputs an edge map directly because it is made to learn edges in an end-to-end fashion. This all-encompassing method guarantees that the model takes into account the picture’s context in its edge detection process, resulting in edge identification that is more precise and consistent across the board. HED processes data at several scales to efficiently capture both fine and coarse features. For the purpose of identifying edges of objects that differ in size, shape, and texture within a single image, multi-scale learning is essential.

**Figure 3:**
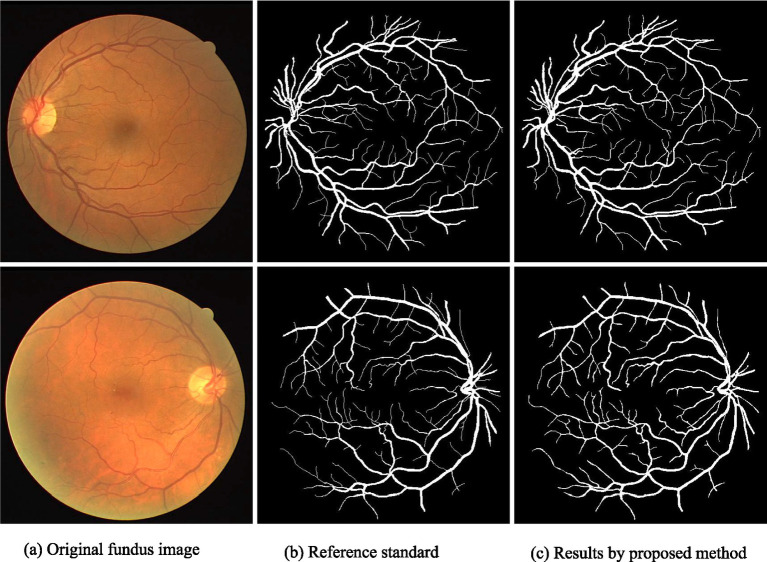
Result analysis using DRIVE datasets.

**Figure 4:**
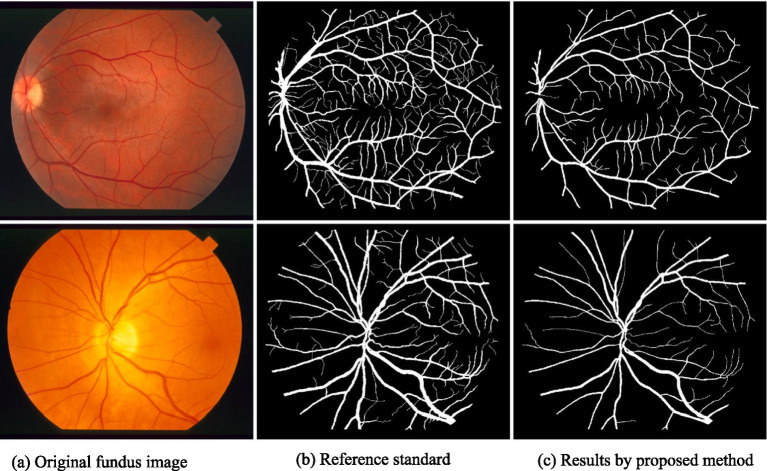
Result analysis using STARE dataset.

Figures 3, 4 demonstrate the outcome analysis utilizing the Drive dataset and the STARE dataset, respectively. In the context of our research, it appears that both DRIU and HED were evaluated as part of the retinal vessel segmentation methods, along with VGAN and other techniques. These models were likely used to provide a comparative analysis of different segmentation approaches in terms of their performance metrics, such as Dice coefficient, accuracy, F1 score, sensitivity, specificity, ROC AUC, and Precision-Recall AUC.

### 4.1 Quantitative evaluation

We employed a range of performance metrics to quantitatively assess the effectiveness of the VGAN model in retinal vessel segmentation. Tables 2, 3 summarizes the results obtained on both the STARE and DRIVE datasets after training the VGAN model for a specified number of epochs. We evaluated our model’s performance using the Intersection over Union (IoU) metric. The IoU is a standard metric for measuring the accuracy of object detection models. Our results indicated that the IoU values were consistently high across the datasets, demonstrating the robustness of our model.

**Table 2:** Performance analysis for STARE dataset.

Epoch	Dice coefficient (DSC)	F1 Score	Accuracy	Sensitivity	Specificity	ROC-AUC	Precision-recall AUC	IoU
2	0.752	0.760	0.852	0.689	0.927	0.824	0.754	0.602564
4	0.764	0.784	0.864	0.694	0.932	0.846	0.762	0.618123
6	0.778	0.798	0.878	0.716	0.938	0.859	0.774	0.636661
8	0.796	0.812	0.884	0.729	0.942	0.871	0.796	0.636661
10	0.804	0.829	0.891	0.746	0.948	0.882	0.809	0.672241
12	0.807	0.831	0.897	0.748	0.949	0.884	0.811	0.676446
14	0.809	0.833	0.899	0.750	0.950	0.886	0.818	0.679261
16	0.812	0.839	0.902	0.752	0.951	0.888	0.823	0.683502
18	0.817	0.842	0.904	0.754	0.952	0.890	0.825	0.690617
20	0.819	0.844	0.906	0.756	0.952	0.892	0.828	0.693480
22	0.823	0.846	0.907	0.758	0.953	0.894	0.832	0.699235
24	0.826	0.848	0.909	0.761	0.953	0.896	0.834	0.703578
26	0.829	0.851	0.911	0.764	0.954	0.898	0.836	0.707942
28	0.831	0.853	0.913	0.768	0.955	0.901	0.838	0.710864
30	0.832	0.855	0.915	0.770	0.955	0.903	0.840	0.712329
32	0.833	0.857	0.917	0.773	0.956	0.905	0.842	0.713796
34	0.835	0.859	0.923	0.775	0.957	0.907	0.844	0.716738
36	0.838	0.861	0.929	0.781	0.959	0.909	0.846	0.721170
38	0.840	0.863	0.930	0.786	0.962	0.911	0.847	0.724138
40	0.842	0.866	0.941	0.793	0.969	0.914	0.849	0.727116
42	0.844	0.869	0.949	0.795	0.971	0.916	0.850	0.730104
44	0.846	0.871	0.953	0.797	0.975	0.918	0.851	0.733102
46	0.849	0.873	0.969	0.804	0.979	0.920	0.853	0.737619
48	0.851	0.875	0.978	0.811	0.984	0.922	0.855	0.740644
50	0.854	0.876	0.982	0.817	0.986	0.924	0.857	0.745201

**Table 3:** Performance analysis for DRIVE dataset.

Epoch	Dice coefficient (DSC)	F1 Score	Accuracy	Sensitivity	Specificity	ROC-AUC	Precision-recall AUC	IoU
2	0.724	0.736	0.845	0.660	0.918	0.792	0.713	0.570020
4	0.732	0.749	0.849	0.678	0.920	0.808	0.728	0.581400
6	0.745	0.754	0.856	0.684	0.922	0.824	0.739	0.599462
8	0.756	0.768	0.861	0.699	0.926	0.837	0.754	0.614035
10	0.763	0.787	0.874	0.712	0.928	0.841	0.766	0.624547
12	0.767	0.789	0.876	0.716	0.929	0.843	0.768	0.629178
14	0.769	0.791	0.878	0.724	0.930	0.846	0.772	0.631656
16	0.770	0.794	0.880	0.728	0.931	0.849	0.775	0.633212
18	0.773	0.796	0.882	0.731	0.932	0.851	0.777	0.636871
20	0.777	0.798	0.883	0.734	0.933	0.853	0.779	0.641533
22	0.779	0.800	0.885	0.739	0.934	0.855	0.782	0.644468
24	0.782	0.802	0.887	0.741	0.935	0.857	0.784	0.648257
26	0.786	0.805	0.889	0.745	0.936	0.859	0.786	0.653485
28	0.789	0.808	0.891	0.747	0.937	0.861	0.789	0.657477
30	0.790	0.811	0.893	0.752	0.938	0.863	0.791	0.659135
32	0.793	0.814	0.895	0.757	0.939	0.865	0.794	0.663021
34	0.795	0.819	0.898	0.759	0.942	0.867	0.796	0.665993
36	0.798	0.822	0.901	0.762	0.947	0.869	0.798	0.669688
38	0.801	0.824	0.918	0.768	0.951	0.870	0.801	0.673366
40	0.805	0.829	0.924	0.772	0.958	0.872	0.804	0.678894
42	0.809	0.831	0.936	0.774	0.964	0.874	0.806	0.683927
44	0.811	0.837	0.948	0.776	0.969	0.876	0.812	0.687160
46	0.816	0.839	0.953	0.779	0.972	0.878	0.814	0.693651
48	0.819	0.842	0.964	0.781	0.979	0.880	0.817	0.693678
50	0.821	0.845	0.970	0.783	0.984	0.882	0.819	0.696154

The comparison of performance analysis utilizing the STARE and DRIVE datasets is shown in Table 4. The proposed network surpassed all previous research in terms of retinal vessel segmentation, as demonstrated in Table 5.

**Table 4:** Comparison of performance analysis using datasets.

References	STARE dataset			DRIVE dataset		
	Sensitivity	Specificity	Accuracy	Sensitivity	Specificity	Accuracy
Jiang et al. (25)	–	–	0.9009	–	–	0.8911
You et al. (26)	0.7260	0.9756	0.9497	0.7410	0.9751	0.9434
Fu et al. (1)	0.7140	–	0.9545	0.7294	–	0.9470
Staal (27)	0.6970	–	0.9516	0.7345	–	0.9443
Marin et al. (28)	0.6944	0.9819	0.9526	0.7067	0.9801	0.9452
Li et al. (29)	–	–	0.9745	–	–	0.9658
Proposed study	0.8174	0.9862	0.9827	0.7834	0.9846	0.9709

**Table 5:** Performance comparison with GAN-based and non-GAN-based methods.

Model variant	Dice coefficient (DSC)	F1 score	Accuracy	Sensitivity	Specificity	ROC-AUC	Precision-recall AUC	IoU
Baseline (Regular 𝐿𝑎𝑑𝑣)	0.82	0.81	0.94	0.80	0.95	-	-	0.695
Proposed 𝐿^∗^_𝑎𝑑𝑣_	0.821	0.845	0.970	0.783	0.984	0.882	0.819	0.696
U-Net (Non-GAN)	0.76	0.78	0.88	0.72	0.91	0.83	0.75	0.613
SegNet (Non-GAN)	0.74	0.77	0.87	0.71	0.90	0.82	0.73	0.587
Pix2Pix (GAN-based)	0.80	0.82	0.93	0.78	0.94	0.87	0.80	0.667
CycleGAN (GAN-based)	0.79	0.81	0.92	0.77	0.93	0.86	0.78	0.653

### 4.2 Qualitative evaluation

**Figure 5:**
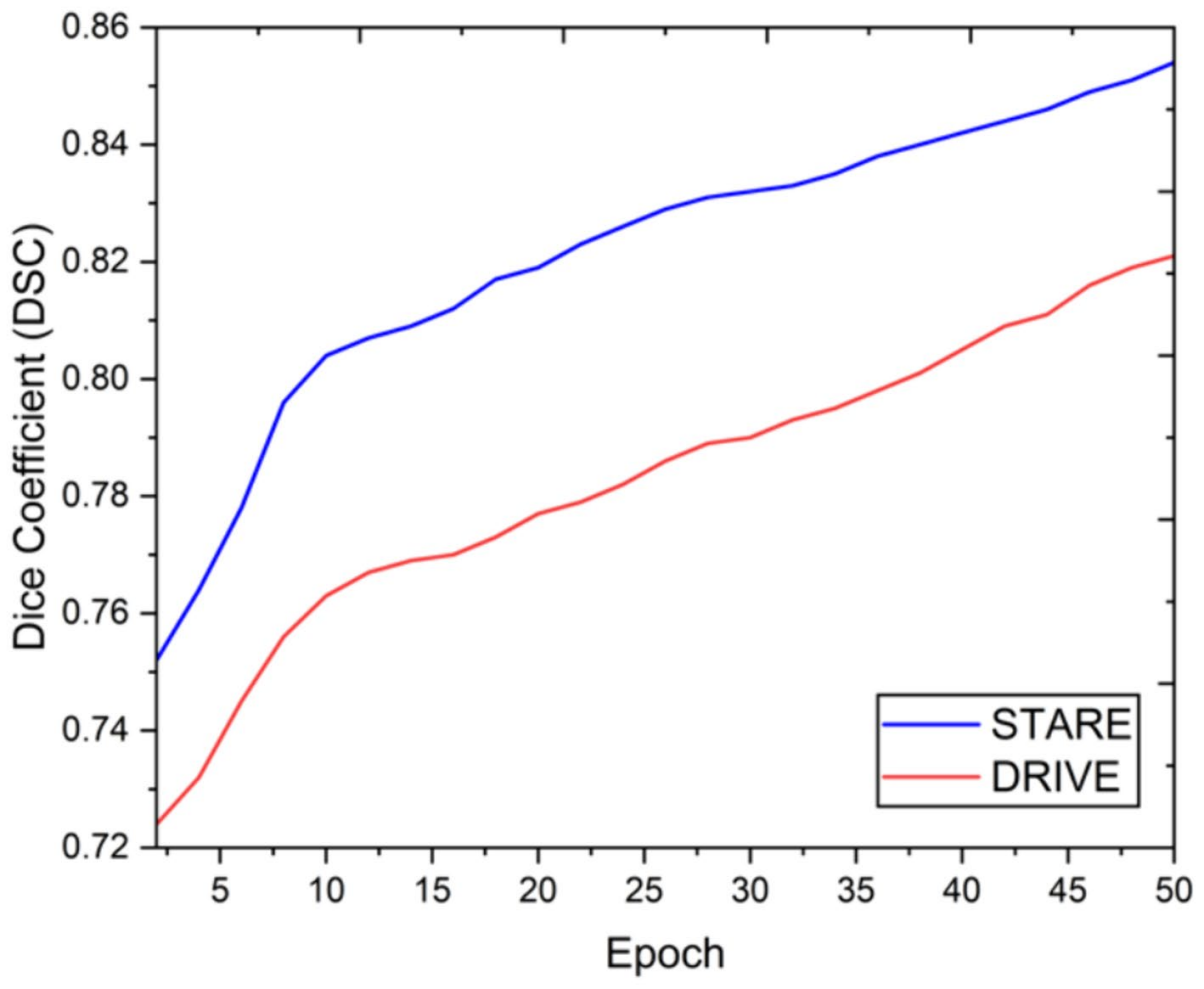
Epoch vs. dice coefficient.

**Figure 6:**
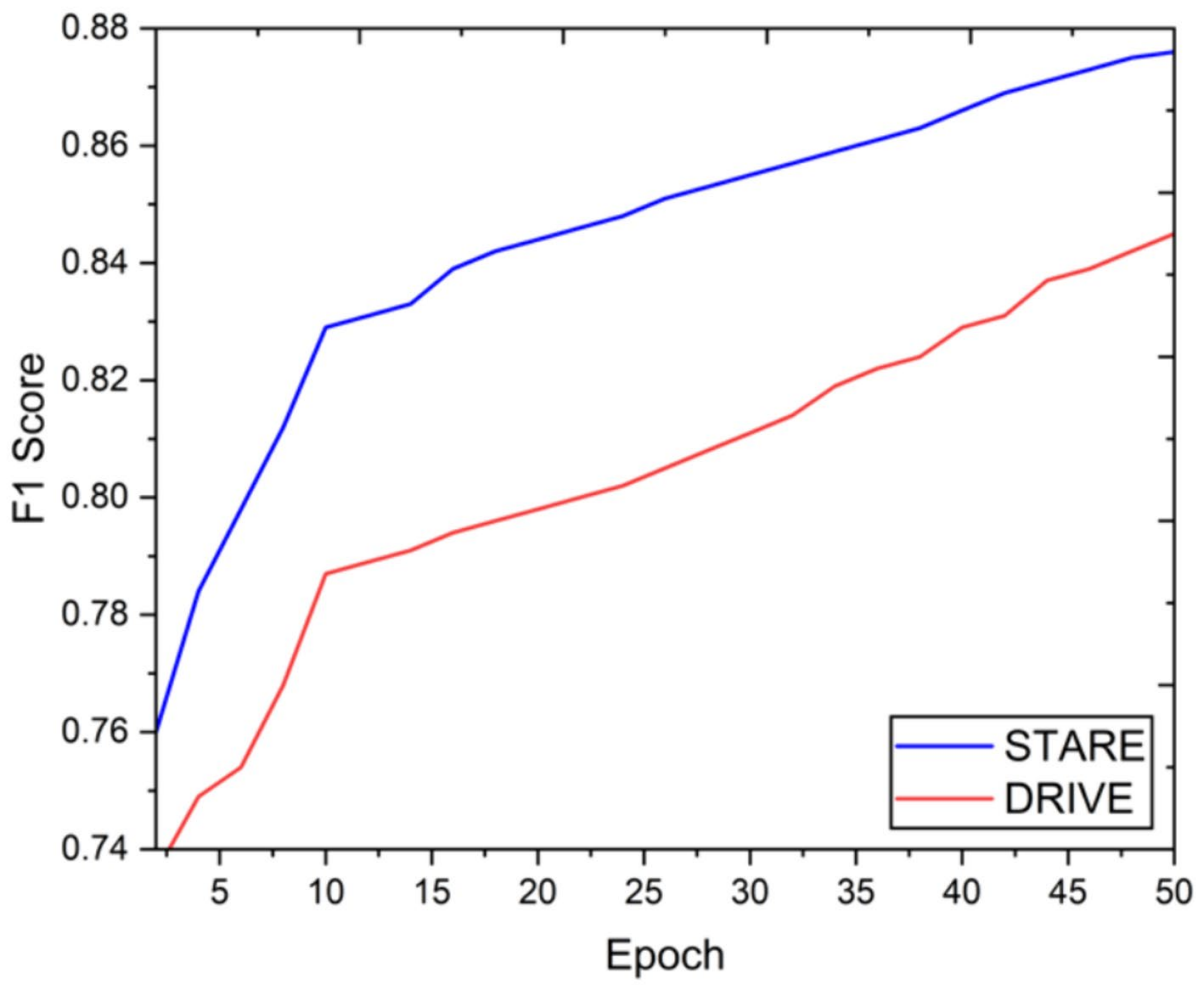
Epoch vs. F1 score.

**Figure 7:**
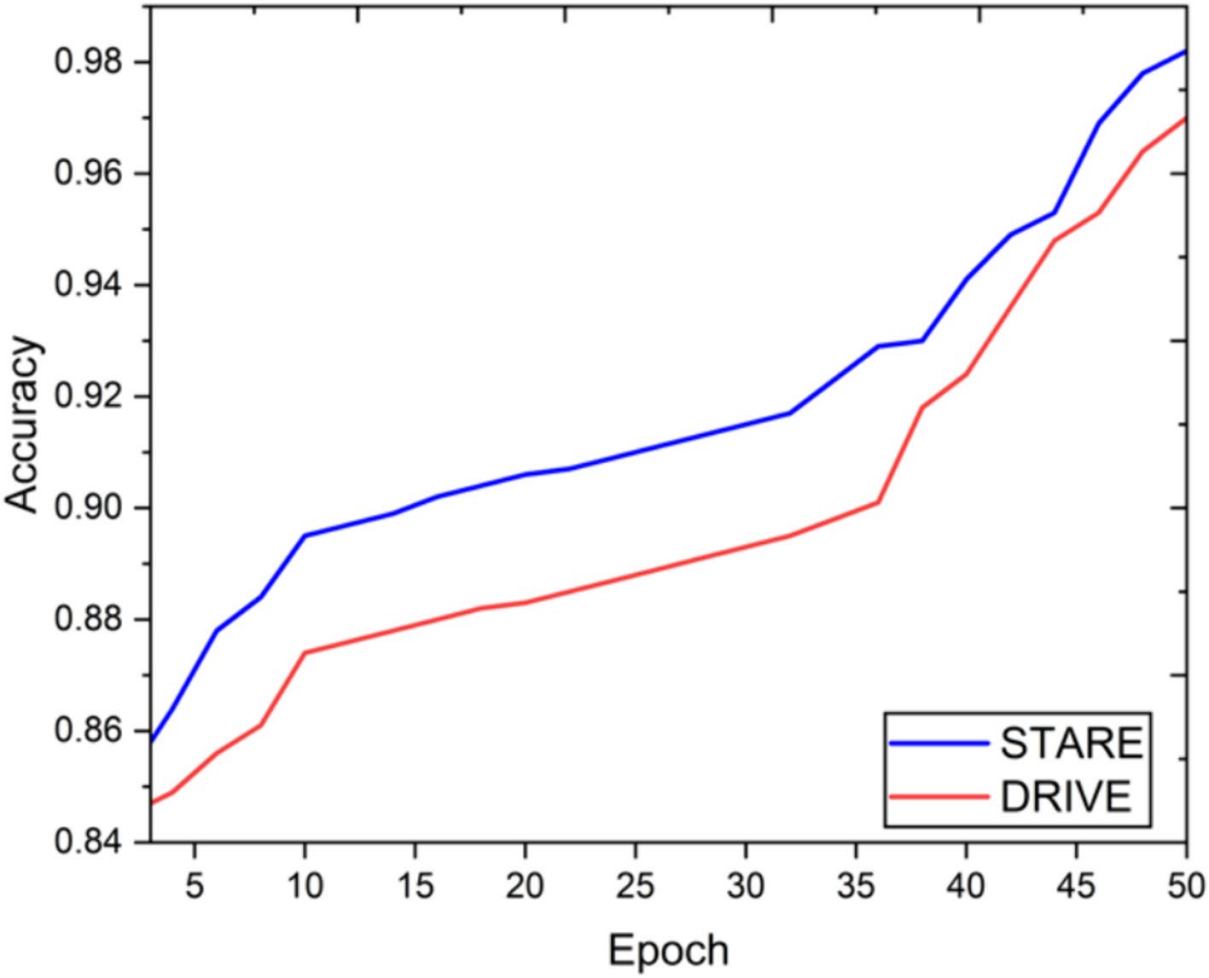
Epoch vs. accuracy.

**Figure 8:**
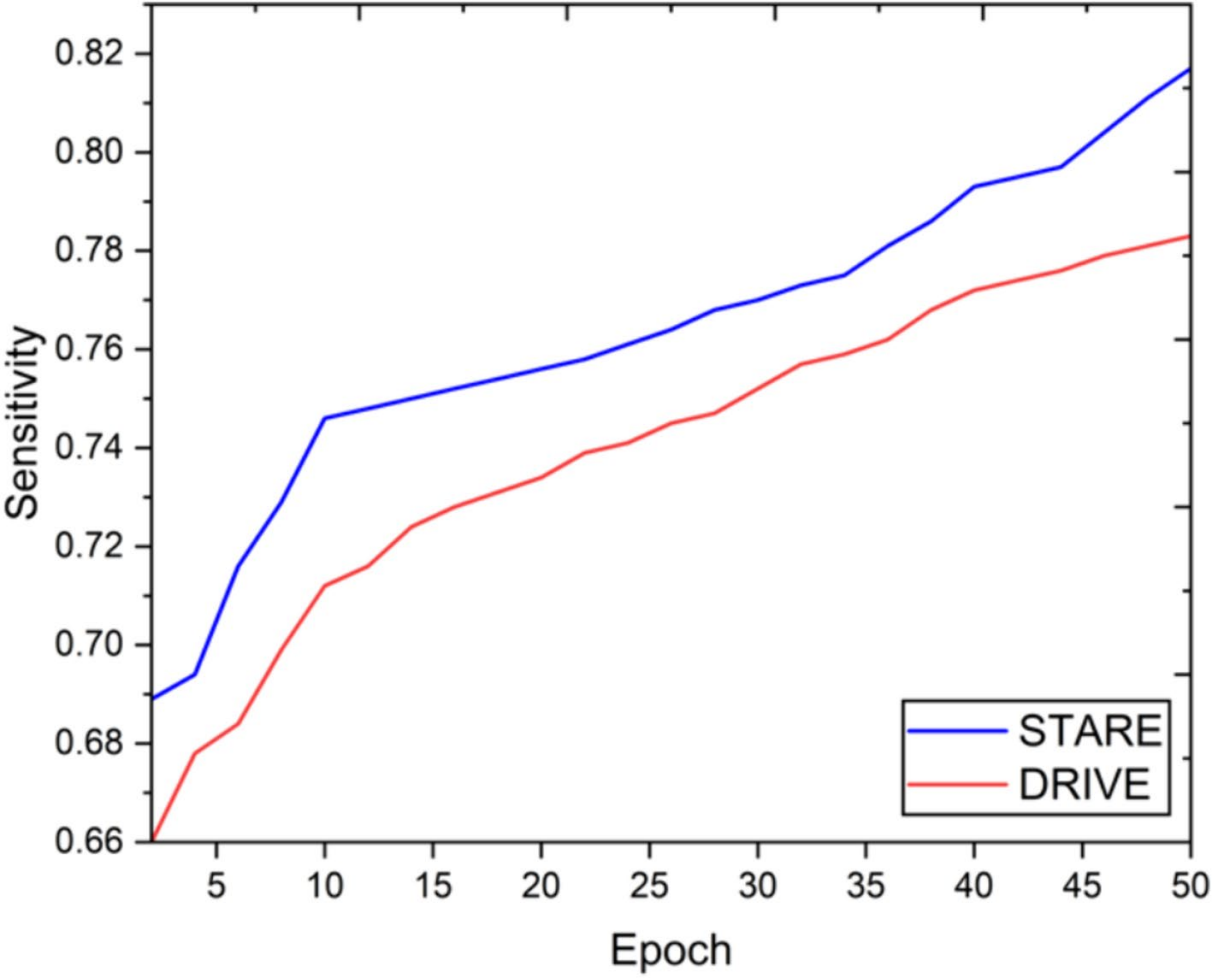
Epoch vs. sensitivity.

**Figure 9:**
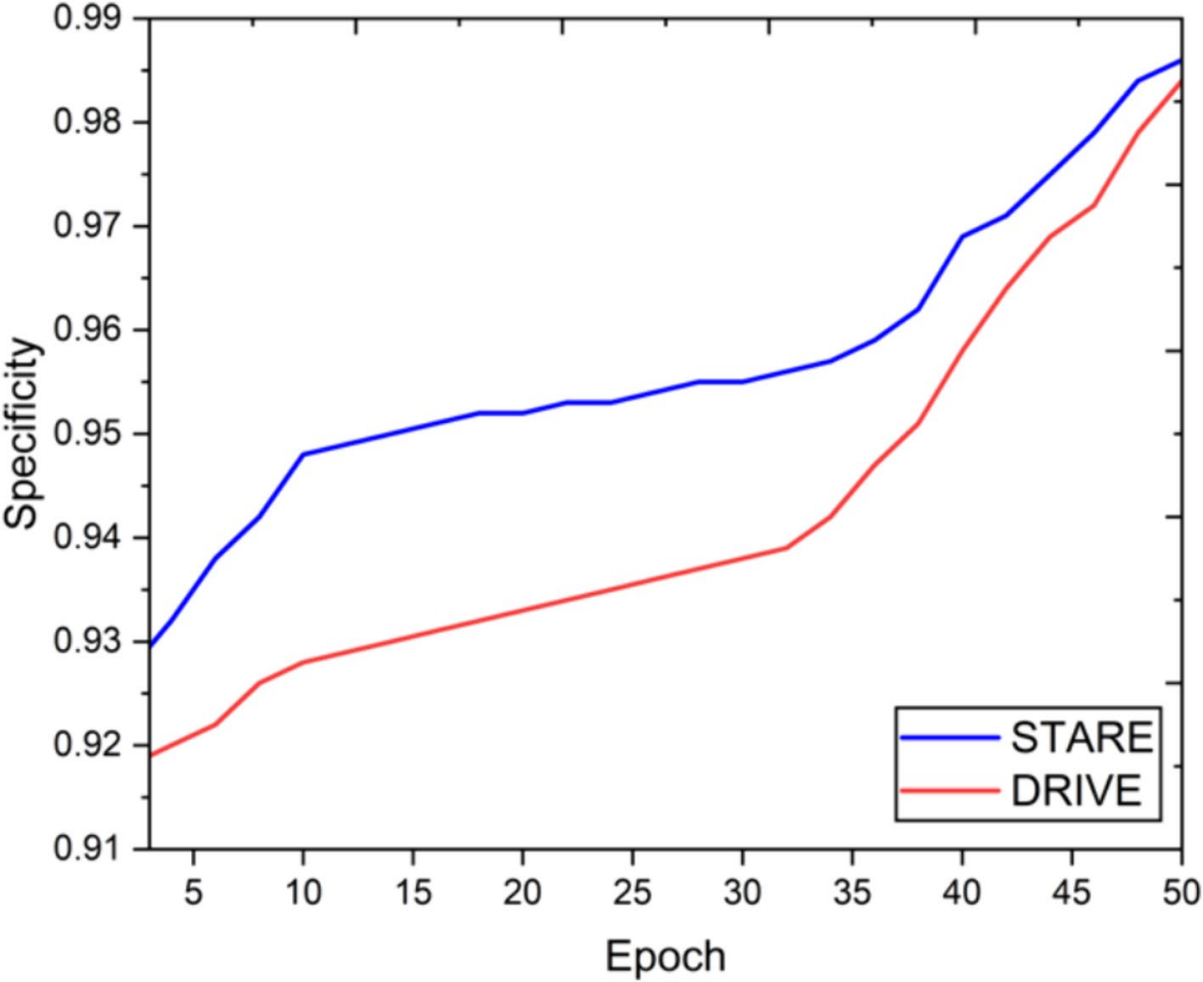
Epoch vs. specificity.

**Figure 10:**
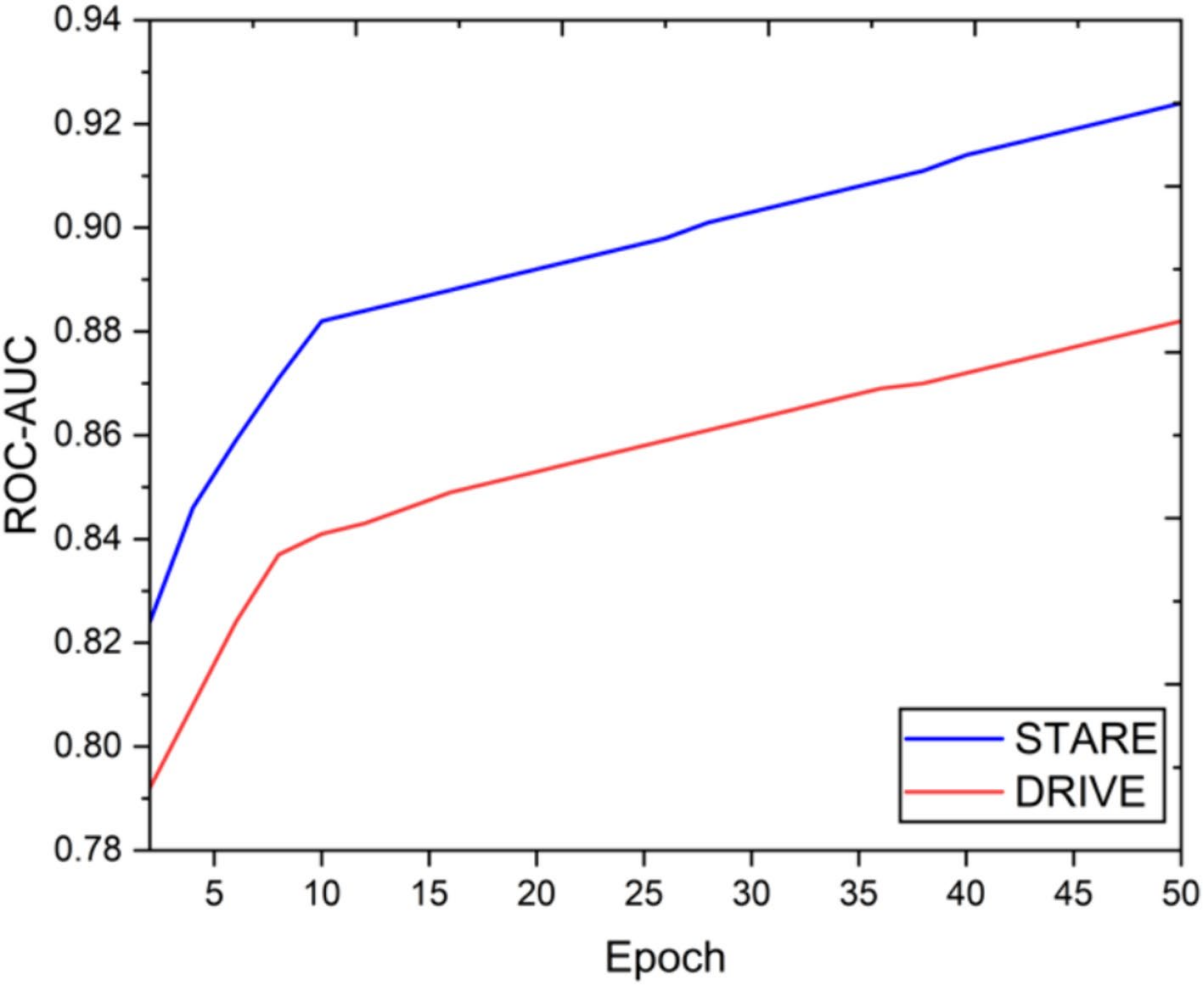
Epoch vs. ROC-AUC.

**Figure 11:**
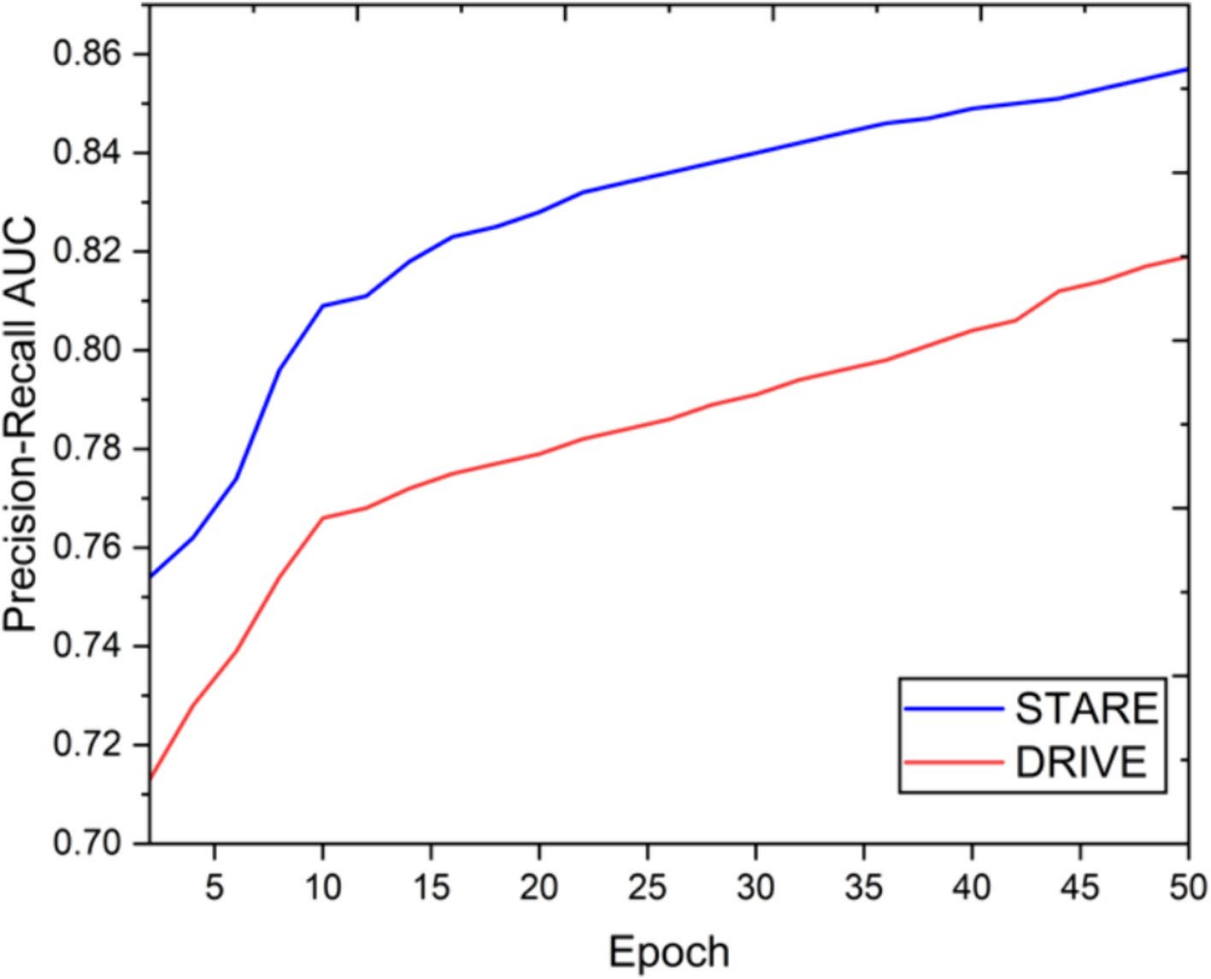
Epoch vs. precision-recall AUC.

Visual inspection of the segmentation results further demonstrates the efficacy of the VGAN model. Figures 5–11 showcases the results of retinal fundus images from both the STARE and DRIVE datasets, along with their corresponding ground truth vessel masks and the vessel masks generated by the VGAN model. The visually consistent alignment between the ground truth and generated masks validates the ability of VGAN to capture intricate vessel structures. The experimental outcomes of epoch vs. dice coefficient, F1 score, accuracy, sensitivity, specificity, ROC-AUC and precision-recall AUC are shown in Figures 5–11.

### 4.3 Discussion

The achieved results underline the remarkable potential of the VGAN architecture for retinal vessel segmentation. The consistently high Dice coefficients, F1 scores, and accuracy values across epochs demonstrate the model’s ability to accurately segment retinal vessels (21, 22). Notably, the increasing trend in performance metrics with the number of training epochs showcases the model’s capacity to improve over time.

The qualitative assessment of the generated vessel masks also reveals the VGAN’s ability to produce visually accurate segmentations that closely resemble ground truth annotations. This aligns with our initial hypothesis that leveraging the power of Variational GANs can significantly enhance the quality of retinal vessel segmentation.

However, like any deep learning approach, VGAN may encounter challenges related to overfitting, generalization, and convergence. Fine-tuning hyperparameters and exploring advanced augmentation techniques could potentially lead to even more robust and stable results (23, 24).

## 5 Conclusion

In this study, we introduced the VGAN model for retinal vessel segmentation and demonstrated its remarkable performance on the STARE and DRIVE datasets. The combination of variational autoencoder and adversarial training led to accurate vessel segmentations, as evidenced by both quantitative metrics and qualitative visual assessments. The VGAN model holds great promise for advancing retinal image analysis, contributing to early disease detection and clinical decision-making in ophthalmology. The success of VGAN in retinal vessel segmentation encourages further research into leveraging generative adversarial networks for medical image analysis, potentially unlocking new avenues for enhancing the accuracy and reliability of diagnostic tools. While our current study highlights the potential of VGAN for retinal vessel segmentation, there are several avenues for future exploration. Investigating novel loss functions, architecture variations, and multi-domain adaptation techniques could further enhance the model’s robustness. Additionally, extending the VGAN framework to handle pathological cases and collaborating with domain experts could yield valuable insights for real-world clinical applications.

## Data Availability

The original contributions presented in the study are included in the article/supplementary material, further inquiries can be directed to the corresponding author.
